# Efficacy and safety of alirocumab in patients with hypercholesterolemia not adequately controlled with non-statin lipid-lowering therapy or the lowest strength of statin: ODYSSEY NIPPON study design and rationale

**DOI:** 10.1186/s12944-017-0513-7

**Published:** 2017-06-17

**Authors:** Tamio Teramoto, Akira Kondo, Arihiro Kiyosue, Mariko Harada-Shiba, Yasushi Ishigaki, Kimimasa Tobita, Yumiko Kawabata, Asuka Ozaki, Marie T. Baccara-Dinet, Masataka Sata

**Affiliations:** 1 Teikyo Academic Research Center, Teikyo University, 2-11-1, Kaga, Itabashi-ku, Tokyo, 173-8605 Japan; 2 Asia Pacific Development, R&D, Sanofi, Tokyo, Japan; 3 Department of Cardiovascular Medicine, Graduate School of Medicine, University of Tokyo, Tokyo, Japan; 4 Department of Molecular Innovation in Lipidology, National Cerebral and Cardiovascular Center Research Institute, Osaka, Japan; 5 Division of Diabetes and Metabolism, Department of Internal Medicine, Iwate Medical University, Iwate, Japan; 6 Sanofi Japan, Tokyo, Japan; 7 PCSK9 Development and Launch Unit, Sanofi, Montpellier, France; 8 Department of Cardiovascular Medicine, Institute of Biomedical Sciences, Tokushima University Graduate School, Tokushima, Japan

**Keywords:** Alirocumab, Hypercholesterolemia, Statin, Statin intolerance, Cardiovascular risk, PCSK9 inhibitor, Lipids

## Abstract

**Background:**

Statins are generally well-tolerated and serious side effects are infrequent, but some patients experience adverse events and reduce their statin dose or discontinue treatment altogether. Alirocumab is a highly specific, fully human monoclonal antibody to proprotein convertase subtilisin/kexin type 9 (PCSK9), which can produce substantial and sustained reductions of low-density lipoprotein cholesterol (LDL-C).

**Methods:**

The randomized, double-blind, placebo-controlled, parallel-group, phase 3 ODYSSEY NIPPON study will explore alirocumab 150 mg every 4 weeks (Q4W) in 163 Japanese patients with hypercholesterolemia who are on the lowest-strength dose of atorvastatin (5 mg/day) or are receiving a non-statin lipid-lowering therapy (LLT) (fenofibrate, bezafibrate, ezetimibe, or diet therapy alone). Hypercholesterolemia is defined as LDL-C ≥ 100 mg/dL (2.6 mmol/L) in patients with heterozygous familial hypercholesterolemia or non-familial hypercholesterolemia with a history of documented coronary heart disease, or ≥120 mg/dL (3.1 mmol/L) in patients with non-familial hypercholesterolemia classified as primary prevention category III (i.e. high-risk patients). During the 12-week double-blind treatment period, patients will be randomized (1:1:1) to receive alirocumab subcutaneously (SC) 150 mg Q4W alternating with placebo for alirocumab Q4W, or alirocumab 150 mg SC every 2 weeks (Q2W), or SC placebo Q2W. The primary efficacy endpoint is the percentage change in calculated LDL-C from baseline to week 12. The long-term safety and tolerability of alirocumab will also be investigated.

**Discussion:**

The ODYSSEY NIPPON study will provide insights into the efficacy and safety of alirocumab 150 mg Q4W or 150 mg Q2W among Japanese patients with hypercholesterolemia who are on the lowest-strength dose of atorvastatin, or are receiving a non-statin LLT (including diet therapy alone).

**Trial registration:**

ClinicalTrials.gov number: NCT02584504

## Background

Lowering low-density lipoprotein cholesterol (LDL-C) levels is a cornerstone for the prevention of cardiovascular disease, the second leading cause of death in Japan [1]. The Japan Atherosclerosis Society (JAS) guidelines recommend lowering LDL-C to <100 mg/dL (2.6 mmol/L) in patients with established cardiovascular disease, to <100 mg/dL (or by 50% or more) in heterozygous familial hypercholesterolemia (heFH), and to <120 mg/dL (3.1 mmol/L) in patients classified as category III in primary prevention [2]. Patients in category III are dyslipidaemic individuals with diabetes mellitus, non-cardiogenic cerebral infarction, or peripheral artery disease, and their 10-year absolute risk of coronary artery disease death is ≥2%. The category also includes dyslipidaemic patients with low high-density lipoprotein cholesterol (<40 mg/dL), and/or family history of premature coronary artery disease, and/or impaired glucose tolerance, and their 10-year absolute risk of coronary artery disease death is ≥0.5% and <2% [2].

Statins substantially reduce the risk of cardiovascular morbidity and mortality [3] and are the preferred treatment option for lowering LDL-C [2, 4, 5]. They are generally well-tolerated and serious side effects are infrequent [6, 7], but some patients experience statin-associated effects [6, 8–14] that result in dose reductions or discontinuation of treatment [15].

Asian patients often show an increased response to drug therapy when compared with Western populations, leading to lower recommended doses of therapeutic drugs [16]. The starting dose of atorvastatin is 10 mg/day in both Japan and the United States, but the maximum approved dose is 40 mg/day in Japan versus 80 mg/day in the United States. Additionally, a 5 mg/day dose is available in Japan. Whereas concerns regarding an increased risk of statin-associated side effects relative to Western populations appear unfounded [16], many Japanese patients still receive a lower dose of statin [17] and a small percentage are receiving non-statin lipid-lowering therapy (LLT) alone [18]. The choice of LLT is also limited because combination therapy comprising a fibrate and a statin – which is associated with an increased risk of rhabdomyolysis [19, 20] – should be used with caution, and avoided among patients with renal disorders [21]. Epidemiological studies have shown that a substantial proportion of treated high-risk Japanese patients do not achieve the JAS LDL-C therapeutic targets [18, 22, 23]. An alternative treatment approach is therefore necessary in patients who are unable to tolerate statins and are not adequately controlled with non-statin LLTs or who are on the lowest-strength dose of statins.

Alirocumab is a highly specific, fully human monoclonal antibody to proprotein convertase subtilisin/kexin type 9 (PCSK9), which plays a key role in low-density lipoprotein receptor degradation. As monotherapy or in combination with other LLT, alirocumab reduced LDL-C by 44–72% at doses of 75–150 mg every 2 weeks (Q2W) in patients with hypercholesterolemia, including heFH [24–32]. The combination of a standard or higher doses of statin plus alirocumab resulted in persistent reductions in LDL-C [24, 26, 29, 32] when administered Q2W, but these reductions were not fully sustained throughout the dosing interval in some patients when alirocumab was administered every 4 weeks (Q4W) [24, 26]. This response is likely due to a statin-induced increase in PCSK9 concentrations [33], leading to greater target-mediated clearance of alirocumab and a reduced duration of effect [34]. The oral LLTs ezetimibe and fenofibrate also appear to increase PCSK9 concentrations in patients with elevated LDL-C, resulting in increased clearance of alirocumab, but the effect is much smaller than that observed with statins [34].

The ODYSSEY NIPPON study will explore the alirocumab 150 mg Q4W regimen in Japanese patients with hypercholesterolemia who are on the lowest-strength dose of atorvastatin (5 mg/day) or are receiving a non-statin LLT (including diet therapy alone). This group represents patients who could potentially benefit from the addition of a PCSK9 inhibitor to their current LLT regimen. The primary objective is to demonstrate the reduction of LDL-C by alirocumab 150 mg Q4W, or 150 mg Q2W, as add-on to the lowest-strength dose of atorvastatin or non-statin LLT (including diet therapy alone), in comparison with placebo after 12 weeks of treatment. The secondary objectives are to evaluate the effect of the two alirocumab regimens on other lipid variables, the safety and tolerability of alirocumab, the development of anti-alirocumab antibodies, the pharmacokinetic and pharmacodynamic profiles of alirocumab, and the long-term safety in patients receiving open-label alirocumab.

## Methods

ODYSSEY NIPPON is a randomized, double-blind, placebo-controlled, parallel-group phase 3 study in 163 patients recruited at 31 sites in Japan. The first patient was enrolled in December 2015 and the last patient was enrolled in October 2016 (http://clinicaltrials.gov/NCT02584504).

### Patients

Patients are eligible to participate if they are aged ≥20 years, have heFH or non-familial hypercholesterolemia (FH), are on the lowest-strength dose of atorvastatin (i.e. 5 mg/day) or are receiving a non-statin therapy (i.e. fenofibrate, bezafibrate, ezetimibe, or diet therapy alone), and have elevated LDL-C values according to the JAS guidelines [2]. Eligible patients will also have one or more documented reason that can explain why statin therapy is not appropriate or why the lowest strength of statin dose should not be increased (e.g. treatment-related side effects, treatment with a CYP3A4 inhibitor, hepatic dysfunction/disease, renal dysfunction, hypothyroidism, glucose intolerance, diabetic nephropathy, older age and low body weight [body mass index <18.5 kg/m^2^], and physician decision). Patients on combination LLT are not eligible for inclusion.

HeFH will be diagnosed by genotyping or clinical criteria [35, 36]. Patients with heFH are eligible regardless of whether they have a history of documented coronary heart disease (i.e. myocardial infarction, unstable angina, percutaneous coronary intervention, coronary artery bypass grafting, or clinically significant coronary heart disease diagnosed by invasive or non-invasive testing such as coronary angiography, stress test using treadmill, stress echocardiography or nuclear imaging). Patients with non-FH must have a history of documented coronary heart disease, or a history of documented diseases or other risk factors classified by the JAS as primary prevention category III (i.e. ischemic stroke, peripheral artery disease, diabetes mellitus, chronic kidney disease, or other risk factors) [2].

Hypercholesterolemia is defined as LDL-C ≥ 100 mg/dL (2.6 mmol/L) in patients with heFH or in patients with non-FH who have a history of documented coronary heart disease, or ≥120 mg/dL (3.1 mmol/L) in patients with non-FH classified as primary prevention category III [2].

### Study design

**Fig. 1:**
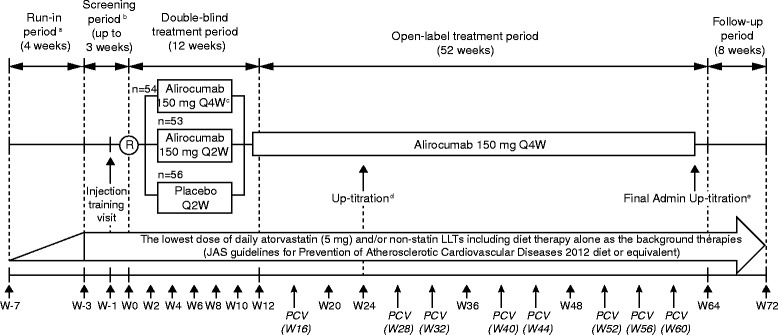
Study design. JAS, Japan Atherosclerosis Society [2]; LDL-C, low-density lipoprotein cholesterol; LLT: lipid-lowering therapy; PCV, phone-call visit; Q2W: every 2 weeks; Q4W: every 4 weeks; R, randomization; W, week. ^a^ As a general rule, patients start the run-in period to reach a stable daily dose of atorvastatin 5 mg or non-statin LLT. ^b^ When patients have received atorvastatin 5 mg/day or non-statin LLT at a stable daily dose for at least 4 weeks, they can skip the run-in period and start the study from the screening period. ^c^ To maintain the double blind, injections of placebo will be conducted at W2, W6, and W10. ^d^ Uptitration to alirocumab 150 mg Q2W at W24 is done only if LDL-C is ≥100 mg/dL or ≥120 mg/dL at W20, depending on risk category. ^e^ Last administration of up-titrated alirocumab 150 mg Q2W at W62. ^f^ The permitted atorvastatin at a dose of 5 mg daily, non-statin LLT, or diet therapy should remain stable (including dose) throughout the study barring exceptional circumstances

The study consists of five periods, illustrated in Fig. 1. During the 4-week run-in, patients will receive stable treatment with atorvastatin 5 mg/day, or non-statin LLTs (ezetimibe, fenofibrate, or bezafibrate), or diet therapy alone, per the physician’s decision. Patients who have already received stable LLT, as described, for ≥4 weeks will be able to enter the screening period directly. During the 3-week screening period, patients or a designated person (e.g. a spouse or relative, if the patient cannot perform the injection) will be trained for self-injection/injection, when necessary. Patients will be instructed to remain on a stable dose of atorvastatin (5 mg/day) or non-statin LLT (ezetimibe, fenofibrate, or bezafibrate) and on a stable diet (JAS guidelines [2] or equivalent) for the study duration, from screening onwards. Other LLTs, including red yeast rice, other fibrates, ethyl icosapentate, and niacin and bile acid sequestrants are not allowed.

During the 12-week double-blind treatment period, patients will be randomized using a 1:1:1 permuted block design to receive alirocumab subcutaneously (SC) 150 mg Q4W alternating with placebo for alirocumab Q4W, or alirocumab 150 mg SC Q2W, or placebo SC for alirocumab Q2W. Injections of study medication (in a 1-mL volume) will be administered by autoinjector into the abdomen, thigh, or outer area of upper arm. Randomization will be stratified according to background statin therapy (Yes/No). “No statin background” will be further stratified by background fibrate/ezetimibe therapy (Yes/No), where ‘Yes’ represents fibrate or ezetimibe, and ‘No’ represents diet therapy alone. All investigational medicinal product (IMP) injections will be conducted by site staff, patients, or designated persons. From week 0 to week 10, all injections will be conducted at the study site by staff or patient/designated person under direct supervision of the staff. From week 12, during the open-label treatment period, all injections can be conducted by patients/designated persons at home if patients agree. Otherwise, they will be conducted by the staff or patients during predefined site visits.

The open-label treatment period will last for 52 weeks, during which all patients will receive alirocumab 150 mg SC Q4W (Fig. 1). At week 24, a dose increase of alirocumab to 150 mg SC Q2W will be automatically conducted through the interactive web response system in patients with heFH or non-FH with documented coronary heart disease if LDL-C is ≥100 mg/dL (2.6 mmol/L) at week 20, and in non-FH category III patients if LDL-C is ≥120 mg/dL (3.1 mmol/L) at week 20. Patients will be followed for 8 weeks after completion of the open-label treatment period.

### Key study endpoints and assessments

The primary efficacy endpoint is the percentage change in calculated LDL-C from baseline to week 12 in the intention-to-treat (ITT) population. The key secondary efficacy endpoints include the change from baseline to week 12 in other atherogenic lipoproteins, listed in Table 1. Lipid variables will be assessed at 0, 4, 8, 10, and 12 weeks (end of double-blind treatment period), and at 20, 24, 36, 48, and 64 weeks (end of open-label treatment period). The laboratory measurement of lipid parameters will be performed by a central laboratory. To maintain the blind, values obtained during the double-blind treatment period will not be communicated to the investigator or sponsor. LDL-C will be calculated using the Friedewald formula [37]. If triglyceride values exceed 400 mg/dL (4.5 mmol/L), the central laboratory will automatically measure LDL-C via the beta-quantification method.

**Table 1:** Key secondary efficacy endpoints (in hierarchical order)

1. The percentage changes in calculated LDL-C from baseline to week 12 in the mITT population, using all LDL-C values during the efficacy double-blind treatment period (on-treatment estimand).
2. The percentage change in calculated LDL-C from baseline to average week 10–12 (ITT estimand).
3. The percentage change in calculated LDL-C from baseline to average week 10–12 (on-treatment estimand).
4. The percentage change in Apo-B from baseline to week 12 (ITT estimand).
5. The percentage change in Apo-B from baseline to week 12 (on-treatment estimand).
6. The percentage change in non-HDL-C from baseline to week 12 (ITT estimand).
7. The percentage change in non-HDL-C from baseline to week 12 (on-treatment estimand).
8. The percentage change in TC from baseline to week 12 (ITT estimand).
9. The proportion of patients reaching LDL-C goal at week 12, i.e. calculated LDL-C < 100 mg/dL (2.6 mmol/L) for heFH or non-FH patients who have a history of documented CHD patients, or LDL-C < 120 mg/dL (3.1 mmol/L) for non-FH patients who have a history of documented diseases or other risk factors classified as primary prevention category III (ITT estimand).
10. The proportion of patients reaching LDL-C goal at week 12, i.e. calculated LDL-C < 100 mg/dL (2.6 mmol/L) for heFH or non-FH patients who have a history of documented CHD patients, or LDL-C < 120 mg/dL (3.1 mmol/L) for non-FH patients who have a history of documented diseases or other risk factors classified as primary prevention category III (on-treatment estimand).
11. The percentage change in lipoprotein(a) from baseline to week 12 (ITT estimand).
12. The percentage change in HDL-C from baseline to week 12 (ITT estimand).
13. The percentage change in fasting triglyceride from baseline to week 12 (ITT estimand).
14. The percentage change in Apo A-1 from baseline to week 12 (ITT estimand).

*Apo* apolipoprotein, *CHD* coronary heart disease, *FH* familial hypercholesterolemia, *HDL-C* high-density lipoprotein cholesterol, *heFH* heterozygous familial hypercholesterolemia, *ITT* intent-to-treat, *LDL-C* low-density lipoprotein cholesterol, *mITT* modified intent-to-treat, *TC* total cholesterol

The endpoints related to evaluation of the safety and tolerability of alirocumab and the development of anti-alirocumab antibodies will be assessed throughout the study. The safety endpoint (comprising adverse events [including adjudicated cardiovascular events], laboratory data, and vital signs) will be analyzed by a central laboratory. The evaluation of anti-alirocumab antibodies will include the antibody status (positive or negative) and antibody titers. Anti-alirocumab antibodies will be determined by the Regeneron Clinical Bioanalysis group (Regeneron Pharmaceuticals Inc. Tarrytown, NY, USA) using a validated non-quantitative, titer-based bridging immunoassay.

### Statistical design and analyses

#### Sample size consideration

Two pairwise comparisons will be performed (alirocumab 150 mg Q4W versus placebo and alirocumab 150 mg Q2W vs placebo) using Bonferroni adjustment to handle multiplicity. A sample size of 38 patients in the ITT population (19 in alirocumab group and 19 in placebo group) was calculated to have 90% power to detect a difference of 30% in mean percent change in calculated LDL-C in any pairwise comparison with a 0.025 two-sided significance level and assuming a common standard deviation of 25%. The total sample size for efficacy would therefore be 57 patients (19 in each of the alirocumab arms and 19 in the placebo arm).

With the sample size consideration for the long-term safety profile, the overall study sample size is 159 patients, allocating 53 patients to alirocumab 150 mg Q4W, 53 patients to alirocumab 150 mg Q2W, and 53 patients to placebo. With this sample size, 100 patients are expected to be exposed to alirocumab for a minimum of 12 months providing that the proportion of dropout is 36%, which is obtained using exponential distribution with the same hazard as that of 30% dropout rate within 12 months. With 100 patients treated with alirocumab for at least 12 months, adverse events with a rate ≥ 0.03 will be detected with 95% probability. Therefore, 159 patients are needed to evaluate both efficacy and safety (163 patients were actually recruited).

#### Efficacy analyses

The randomized population will include all patients who have been allocated to a randomized treatment regardless of whether they received the study medication. The primary efficacy analysis population will be the ITT population, defined as the randomized population who has an evaluable primary efficacy endpoint (i.e. patients with at least one value for calculated LDL-C before the first double-blind dose [i.e. baseline] and at least one value for calculated LDL-C for weeks 4, 8, 10, or 12). The modified ITT (mITT) population is defined as the randomized population who took at least one dose or partial dose of the double-blind injection and had an evaluable primary efficacy endpoint during the efficacy double-blind treatment period, defined as the period from the first double-blind IMP injection up to 21 days after the last double-blind IMP injection. Patients will be analyzed according to the treatment group allocated by randomization.

The primary efficacy endpoints will be analyzed in the ITT population using a mixed-effect model with repeated measures (MMRM) approach. The model will include the fixed categorical effects of treatment group, time point (4, 8, 10, 12 weeks), stratification factor of statin (Yes/No), treatment-by-time-point interaction and statin-by-time-point interaction, as well as the continuous fixed covariates of baseline calculated LDL-C value, and baseline value-by-time-point interaction. This model is used to provide baseline adjusted least square (LS) mean estimates at week 12 for each treatment group with their corresponding standard errors. Each alirocumab group will be compared to the placebo group (at significance level of 2.5%) using appropriate contrasts, and the 97.5% confidence interval of the difference will be provided. Subgroup analyses will be performed to assess the consistency of treatment effect within stratification factor of statin (Yes/No).

Continuous secondary endpoints anticipated to have a normal distribution will be analyzed using the same MMRM model as for the primary endpoint with the continuous fixed covariates of corresponding baseline value and baseline value-by-time-point interaction. Those anticipated to have a non-normal distribution (i.e. lipoprotein[a] and triglycerides) will be analyzed using a multiple imputation approach for handling of missing values followed by robust regression. Binary secondary endpoints will be analyzed using a multiple imputation approach for handling of missing values followed by logistic regression. In the data-dependent case that the logistic regression method is not applicable (e.g. the response rate is zero in one treatment arm and thus the maximum likelihood estimate may not exist), the last observation carried forward approach would be used for handling of missing values and a stratified exact conditional logistic regression would be performed to compare treatment effects.

A hierarchical procedure will be used for each pairwise comparison to control type I error and to handle multiple endpoints. If the primary endpoint analysis is significant at the 2.5% alpha level, the key secondary efficacy endpoints will be tested sequentially at the 0.025 level, using the order defined in Table 1. The hierarchical procedure for comparisons of alirocumab 150 mg Q4W versus placebo and alirocumab 150 mg Q2W versus placebo will be processed separately. Analyses conducted in the open-label treatment period will be exploratory only.

### Safety analysis

The safety analysis will be descriptive. The safety population will comprise the randomized population who received at least one dose or partial dose of study medication, and will be analyzed according to the treatment received.

## Discussion

An unmet medical need exists for additional, efficacious, and well-tolerated therapeutic options in patients with hypercholesterolemia who exhibit an inadequate lipid-lowering response to current therapies or who are unable to take standard or higher doses of statins because of side effects, contraindications, or for other reasons.

The efficacy and safety of alirocumab have been investigated in Japanese populations in phase 1 and 2 studies [38]; their results were confirmed in a 52-week Japanese phase 3 study involving 216 high-risk adults (defined as heFH, non-FH at high cardiovascular risk with coronary disease, or classified as category III) [39] treated with 75 mg Q2W with the potential for titration to 150 mg Q2W at week 12 if the LDL-C target was not met at week 8. At week 24, mean change in LDL-C from baseline was −62.5% in the alirocumab group versus 1.6% in the placebo group (*P* < 0.0001) with a dose increase to 150 mg Q2W in only 1.4% of patients; the reduction was sustained to week 52 [39]. Combined, the results of these studies show that alirocumab significantly reduced LDL-C concentrations versus placebo in high-risk Japanese patients on stable statin therapy with or without other LLTs and was generally well tolerated. The most frequently reported adverse events were nasopharyngitis (45.5% in the alirocumab group versus 36.1% in the placebo group), back pain (12.6% vs 5.6%, respectively), and injection site reaction (12.6% vs 4.2%, respectively).

A randomized, placebo-controlled, phase 1 study – conducted outside of Japan – investigated ascending doses of alirocumab in patients with and without FH (50, 100, 150 mg) [40]. The study included three cohorts: the first involved 21 patients with heFH on atorvastatin therapy; the second cohort included 30 non-FH patients, also on atorvastatin therapy; whereas the third cohort of 10 patients with non-FH were being treated with diet-therapy alone. With the 150 mg dose of alirocumab, 2 weeks after the first administration, the mean difference in percent change in LDL-C from baseline, versus placebo, was at least 46% in all three cohorts. The effects of alirocumab and atorvastatin in lowering LDL-C appeared to be additive, as the mean percent reductions were similar in the three cohorts; however, they were not fully maintained over the dosing period in patients already receiving a statin. The “bounce back” in LDL-C value observed in the statin-treated cohort is likely due to the fact that statins stimulate the production of PCSK9; while this does not alter the LDL-lowering effect of alirocumab, it appears to reduce the duration of action [34]. In contrast, the reduction in LDL-C was consistent over the dosing period in the patients treated with diet-therapy alone, suggesting that alirocumab has a longer duration of action in the absence of a statin.

Ezetimibe and fenofibrate also appear to increase PCSK9 concentrations, but the effect is much less substantial than that with statins [34]. This finding is illustrated in a partial-blinded French study involving 72 patients with LDL-C levels ≥100 mg, randomized to once-daily oral dosing with fenofibrate 160 mg, ezetimibe 10 mg, or placebo, plus alirocumab 150 mg Q4W for 3 months [34]. The mean reductions in LDL-C with alirocumab plus placebo were maintained over the 4-week dosing interval (percent change from baseline: 44.4% at day 57, 47.4% at day 71, and 47.0% at day 85). Whereas greater reductions in LDL-C were achieved with the combination LLT (65.3% with ezetimibe and 66.8% with fenofibrate, versus 48.2% with placebo, at day 71, 2 weeks after the last dose), the PCSK9 levels and the alirocumab clearance rate were increased, and the efficacy was slightly less sustained between drug administrations with the combination LLT (49.6% with ezetimibe and 43.2% with fenofibrate at day 85, 4 weeks after the last dose). The alirocumab 150 mg Q4W regimen could therefore provide ongoing and efficacious reductions in LDL-C over a 4-week dosing interval when administered as monotherapy or in combination with non-statin LLT [34]. This dosing regimen might be a preferred option in some patients.

Two phase 3 studies have evaluated the Q4W alirocumab regimen, in predominantly white populations. ODYSSEY CHOICE II [41] followed a ‘treat-to-target’ dosing strategy, and evaluated the efficacy and safety of alirocumab 150 mg Q4W (with possible adjustment to 150 mg Q2W) in patients with hypercholesterolemia not receiving statin therapy. Most of the study population (90.1%) had a history of intolerable statin-associated muscle symptoms. The alirocumab 150 mg Q4W regimen led to LDL-C reductions from baseline of −41.7% at week 12, suggesting that this strategy may be an additional therapeutic option in Japan for patients with hypercholesterolemia not on statin therapy, and notably for those intolerant of statin treatment. However, this dosing strategy may not provide adequate LDL-C reduction in all patients, for example, those receiving concomitant statin or those with higher baseline LDL-C levels. ODYSSEY CHOICE I investigated the efficacy, long-term safety, and tolerability of an alirocumab 300 mg Q4W regimen, with dose adjustment at week 12 to 150 mg Q2W depending on individual patient response, as add-on to statin therapy or monotherapy in moderate to very high risk patients with hypercholesterolemia [42]. This alirocumab regimen showed statistically significant reductions from baseline in LDL-C in patients receiving and not receiving concomitant statin, with no increase in treatment-emergent adverse events across the study groups. At week 24, a reduction of −52.7% was achieved with alirocumab 300 mg Q4W in patients not receiving statin (14.7% of whom had dose adjustment to 150 mg Q2W) and of −58.8% in patients receiving concomitant statin therapy (19.3% of whom had dose adjustment) [42].

## Conclusions

The data from the ODYSSEY NIPPON study will provide insights into the efficacy and safety of alirocumab 150 mg Q4W or 150 mg Q2W among Japanese patients with hypercholesterolemia who are on the lowest-strength dose of statin, or are receiving a non-statin LLT (including diet therapy alone). This group of patients could potentially achieve lower LDL-C levels with the addition of a PCSK9 inhibitor to their current LLT regimen using a monthly dosing regimen.

## Abbreviations

FH: Familial hypercholesterolemia
HeFH: Heterozygous familial hypercholesterolemia
IMP: Investigational medicinal product
ITT: Intention-to-treat
JAS: Japan Atherosclerosis Society
LDL-C: Low-density lipoprotein cholesterol
LLT: Lipid-lowering therapy
mITT: Modified intention-to-treat
MMRM: Mixed-effect model with repeated measures
PCSK9: Proprotein convertase subtilisin/kexin type 9
Q2W: Every 2 weeks
Q4W: Every 4 weeks
SC: Subcutaneous/subcutaneously
